# Short-term assessment of radial artery grafts with multidetector computed tomography

**DOI:** 10.1186/s13019-021-01465-3

**Published:** 2021-04-17

**Authors:** En Qiao, Yuetang Wang, Jun Yu, Xu Wang, Xinjin Luo, Wei Wang

**Affiliations:** Structural Heart Disease Center, Fuwai Hospital, National Center for Cardiovascular Disease, Chinese Academy of Medical Sciences, Peking Union Medical College, 167 Beilishi Road, Xicheng District, Beijing, 100037 China

**Keywords:** Coronary artery bypass grafting, Radial artery, Coronary artery disease, Multidetector computed tomography

## Abstract

**Background:**

The clinical use of the radial artery (RA) in coronary artery bypass grafting (CABG) is still limited worldwide, although it has been recommended by several guidelines. Multidetector computed tomography (MDCT) is widely used to evaluate graft patency, as invasive coronary angiography could cause potentially serious risks including bleeding, dissection and stroke. This study aims to report the short-term results of the RA in CABG with MDCT.

**Methods:**

The study population consists of 41 consecutive patients undergoing elective CABG with the RA graft between 2017 to 2018, with MDCT performed to evaluate graft patency during follow-up, and target vessels for the RA were non-left anterior descending coronary arteries with > 70% stenosis.

**Results:**

A total of 150 grafts were assessed by MDCT during follow-up (mean, 8.9 ± 5.1 months). MDCT could clearly show the structure and patency of grafts, even for complex coronary artery revascularization. Graft patency of the left internal mammary artery was 92.9% (39/42), with the RA patency of 84.4% (38/45) and the patency of the saphenous vein graft of 81.1% (30/37). And the RA anastomosed to the left coronary artery system might have better patency than the RA anastomosed to the right coronary artery system (25/29, 86.2% vs 13/16, 81.3%, *p* = 0.686).

**Conclusions:**

The short-term patency rate of RA grafts is good, and the RA might be associated with better patency when anastomosed to the left but not the right coronary artery. MDCT could provide excellent visualization of grafts in CABG.

## Background

The radial artery (RA), following the left internal mammary artery (LIMA) and saphenous vein graft (SVG), is the most recently introduced conduit in coronary artery bypass grafting (CABG). However, due to its susceptibility to spasm and the possible harm to the forearm circulation, current studies present an inadequate use of the RA in CABG. A recent report, 2019 Cardiovascular Surgery Outcomes of Fuwai Hospital, showed that less than 5% patients underwent CABG using the RA in our institution in the period 2010 to 2019. Multidetector computed tomography (MDCT), a noninvasive and cheaper diagnostic tool compared with invasive coronary angiography (ICA), has been found to be valuable and reliable in CABG graft evaluation. Moreover, current literature on RA patency remains to be limited, so we carried out this study to evaluate the quality of RA grafts with MDCT.

## Methods

### Patients

From 2017 to 2018, 41 consecutive patients underwent CABG using RA conduits in Fuwai Hospital and had MDCT to assess the quality of bypass conduits during follow-up. The primary indications of RA utilization were an age less than 70 years old and target vessels with > 70% stenosis, primarily on the basis of the work of Tranbaugh et al. [1]. The primary outcome was graft patency, and secondary outcomes included death, myocardial infarction, stroke and repeat revascularization.

### Surgical methods

The Allen test served as the standard of screening RA grafts. The RA was harvested from the nondominant arm, including 40 left RAs and 1 right RA in this study, without bilateral RAs obtained. The forearm was positioned horizontal and abducted to about 80° to the body. A straight skin incision was made from 2 cm inside the wrist fold line to 2 cm beyond the cubital fossa. Then, the forearm fascia was separated, and the RA was exposed and isolated along with the accompanying veins and some adipose tissue. After the patient was systematically heparinized, the distal and proximal ends of the RA were cut in order; a special solution containing verapamil, heparin and papaverine was gently injected into the vessel lumen, and additional bleeding points were located and clipped at the same time. Afterwards, the RA was put into this special solution until used.

### MDCT angiography and image interpretation

Image acquisition was performed using a 128-slince MDCT system (SOMATOM Definition Flash, Siemens Healthcare, Forchheim, Germany). Metoprolol 25-50 mg was administrated to patients orally 1 h before scanning if the heart rate was over 65 beats per minute. A bolus of low-osmosis, high-concentration, non-ionic, iodinated contrast medium (iopromide [Ultravist] 370 mg iodine/ml, Bayer Healthcare, Berlin, Germany) was injected via the antecubital vein through a power injector through an 18–20 gauge needle at a rate of 4 ml/s followed by a saline chaser of 40 ml. The volume of contrast medium was individually determined according to patient weight. Imaging parameters were set as follows: tuber voltage, 100–120 kV; tuber current determined by Care Dose 4D (Siemens); rotation time, 300 ms; collimation, 2x128x0.625 mm; and pitch, 0.15–0.25. To ensure consistency between patients, CT reconstruction was performed using a soft convolution kernel [2] from a data acquisition window centered at 70% of the RR interval. CT images were transferred to the system’s integrated workstation (Syngo.Via, version VB10A, Siemens Healthcare, Erlangen, Germany) for reconstruction, measurement and analysis.

CT images were evaluated by 2 experienced radiologists who were aware of the previous CABG graft type. Each anastomosis was considered as a separate graft. The patency analysis of each graft was performed at the proximal anastomosis or the vessel origin (LIMA), along the free graft body, and at the distal anastomosis. And the graft patency status was classified into 3 descriptive imaging categories: patent (no stenosis or stenosis < 50%), stenosis ≥50%, and occluded. A graft was diagnosed with occlusion if it was not visualized at all or was shown as a stump-like structure.

### Data collection

Patients were followed up at regular intervals in our situation, 3 months, 6 months, 1 year after surgery and yearly thereafter. During follow-up, besides MDCT, blood examinations, chest roentgenogram, electrocardiography and transthoracic echocardiography (TTE) were performed. Myocardial infarction, stroke, repeat revascularization, mortality and other late complications from any cause were obtained from telephone interviews and postoperative clinical consultations.

### Statistical analysis

All results were stored with Microsoft Excel [Microsoft, Redmond, Wash], and all statistical analyses were conducted using SPSS software, version 24 [IBM, Armonk, NY]. Continuous variables were reported as mean ± standard deviation, and categorical variables were reported as frequencies and percentages. For the comparison of graft patency, statistical analysis was performed using the Fisher’s exact test. A *p* value of less than 0.05 was considered to indicate statistical difference.

## Results

### Clinical status of patients

From October 2017 to August 2018, 41 consecutive patients (male/female, 39/2) underwent CABG in our hospital, with the mean age of 49.6 ± 6.2 years. Thirty-nine patients had dyslipidemia, with 23 and 14 cases diagnosed with hypertension and diabetic mellitus respectively. Twenty-eight patients had three-vessel disease; 12 and 1 patients had two-vessel and one-vessel disease respectively. The TTE showed left ventricular ejection fraction (LVEF) was 62.2 ± 5.9% and left ventricular end diastolic dimension (LVEDD) was 49.2 ± 6.3 mm. All operations were performed by the same surgical team, with on-pump CABG in 29 (70.7%) cases and off-pump in 12 (29.3%), and no emergency surgeries were performed. The mean cardiopulmonary bypass time was 91.9 ± 64.0 min, and the mean cross-clamp time was 67.0 ± 47.7 min. (Table 1) Calcium-channel blockers (CCBs) were prescribed for all patients for the first year after operation and antiplatelet agents were prescribed for long term; CCB agents were used in 36 (87.8%) cases and all cases continued to take antiplatelet drugs at the latest follow-up (mean, 8.9 ± 5.1 months). During the follow-up period, there were no recurrent myocardial infarctions, cerebral infarctions, mortalities or repeat revascularizations. Two patients developed paresthesia in the left hand before discharge from the hospital; 1 case of them felt numbness at the part between the thumb and the index finger, and another case felt numbness at the back of the thumb. At the latest follow-up, numbness at the part between the thumb and the index finger on 1 case disappeared, while another case still maintained the original hand symptom, and the incidence of paresthesia in the early postoperative period was 2.4%.

**Table 1:** Preoperative and intraoperative characteristics

Characteristic	Results (*N* = 41)
Mean age (years)	49.6 ± 6.2
Male/Female	39/2
Hypertension	23 (56.1%)
Dyslipidemia	39 (95.1%)
Diabetes Mellitus	14 (34.1%)
Creatinine (umol/l)	82.2 ± 13.9
CK-MB (U/l)	4.9 ± 6.1
Smoke	28 (68.3%)
Peripheral vascular disease	4 (9.8%)
On-pump CABG	29 (70.7%)
Preoperative PCI	6 (14.6%)
LVEF (%)	62.2 ± 5.9
LVEDD (mm)	49.2 ± 6.3
Age group	
<50	19 (46.3%)
≥ 50	22 (53.7%)
Coronary vessel disease	
Single	1 (2.4%)
Double	12 (29.3%)
Triple	28 (68.3%)
Total arterial CABG	19 (46.3%)

*CABG* Coronary artery bypass graft, *PCI* Percutaneous coronary intervention, *LVEF* Left ventricular ejection fraction, *LVEDD* Left ventricular end diastolic dimension

### Feasibility of MDCT

**Fig. 1:**
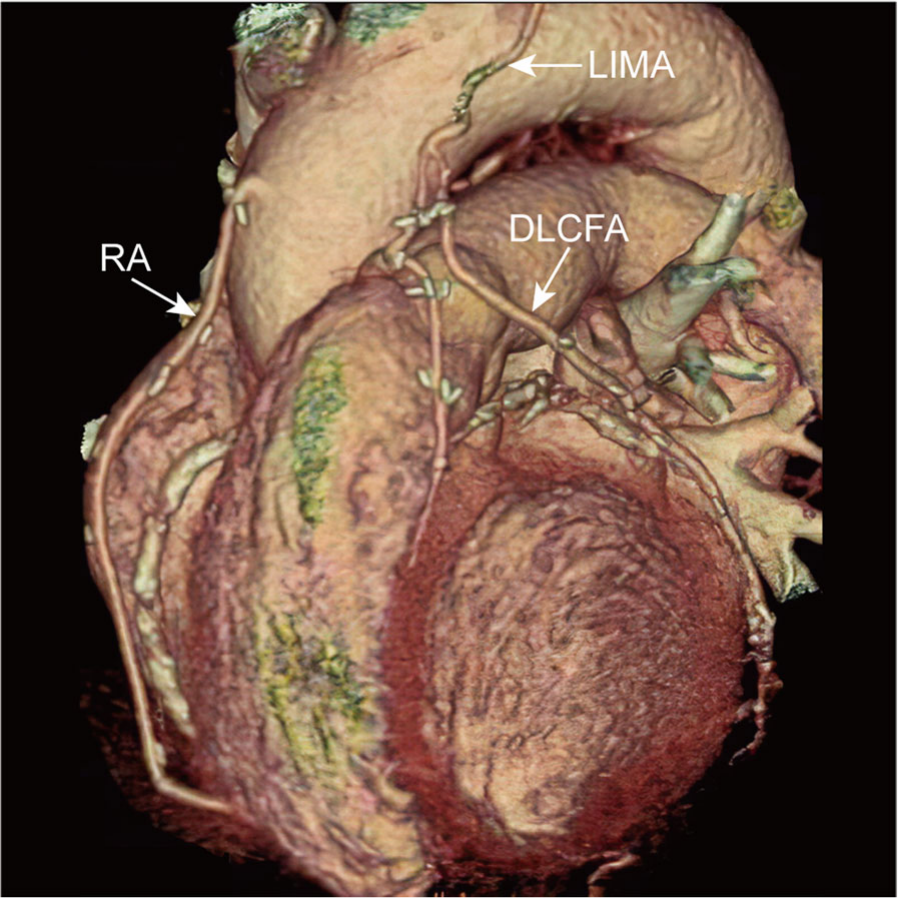
The three-dimensional MDCT image of one patient shows patent LIMA graft to LAD, patent DLCFA graft to OM, and patent RA graft to PDA

**Fig. 2:**
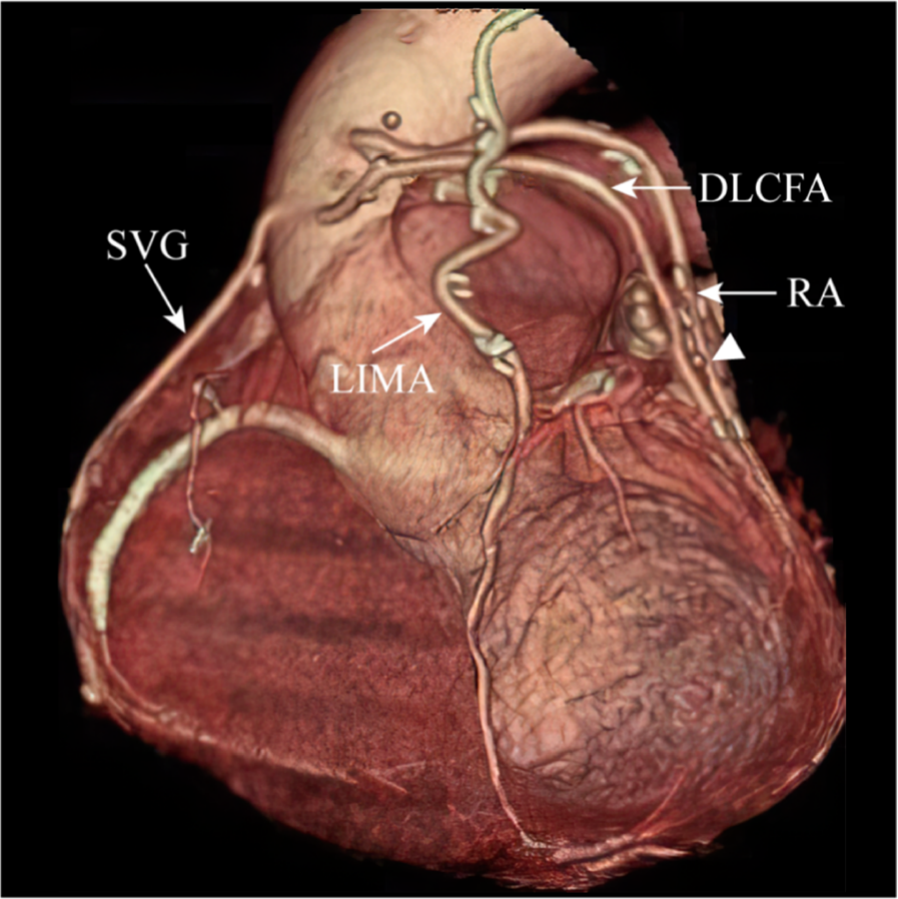
The three-dimensional MDCT image demonstrates patent LIMA graft to LAD, patent DLCFA graft to D, patent SVG graft to PDA, and stenosis RA graft to OM in the distal conduit (arrowhead)

MDCT was performed without complications; all patients agreed to the examination, and no cases complained of any discomfort. Excellent graft images including the anastomoses were obtained in all patients (shown in Figs. 1-2).

### Graft patency

All 41 patients underwent MDCT to evaluate graft patency during follow-up. There were 150 distal anastomoses in total, with the mean of 3.7 (rang 2 to 5) per patient, consisting of 45 RA anastomoses, 42 LIMA anastomoses, 10 right internal mammary artery (RIMA) anastomoses, 16 descending branch of the lateral circumflex femoral artery (DLCFA) anastomoses and 37 SVG anastomoses. Total arterial revascularization was performed in 19 patients, and each anastomosis was regarded as a graft. The LIMA had the highest patency rate (92.9%); the RA had a patency rate of 84.4%, which was slightly better than the SVG (81.1%) and the RIMA (80%). The DLCFA could also be used as an alternative graft conduit for CABG, with the patency of 75%. (Table 2) The RA might obtain better patency than the SVG, when anastomosed to the diagonal branch (D, 90% vs 80%) or posterior descending artery (PDA, 80% vs 75%), while the SVG might obtain slightly better patency than the RA (90% vs 88.2%), when anastomosed to the obtuse marginal branch (OM); however, no statistical difference was observed. (Table 3).

**Table 2:** Patency rates for bypass grafts for the 41 study patients by graft type

Graft type	N anastomoses	N patent	% Patent
LIMA	42	39	92.9
RA	45	38	84.4
SVG	37	30	81.1
RIMA	10	8	80.0
DLCFA	16	12	75.0

*LIMA* Left internal mammary artery, *RA* Radial artery, *SVG* Saphenous vein graft, *RIMA* Right internal mammary artery, *DLCFA* Descending branch of the lateral circumflex femoral artery

**Table 3:** Patency comparison of the RA and non-RA grafts

Target	Graft	Results	N patent	% patent	*p* value
IM					
	RA	2	1	50.0%	NA
	LIMA	2	1	50.0%	1
	SVG	1	1	100.0%	1
D					
	RA	10	9	90.0%	NA
	SVG	10	8	80.0%	1
	DLCFA	7	5	71.4%	0.537
	LIMA	4	4	100.0%	1
OM					
	RA	17	15	88.2%	NA
	SVG	10	9	90.0%	1
	DLCFA	8	6	75.0%	0.57
PDA					
	RA	15	12	80.0%	NA
	SVG	12	9	75.0%	1
	RIMA	4	3	75.0%	1
	DLCFA	1	1	100.0%	1
RCA					
	RA	1	1	100.0%	NA
	RIMA	1	1	100.0%	1

*IM* Intermediate branch, *D* Diagonal branch, *OM* Obtuse marginal branch, *PDA* Posterior descending artery, *RCA* Right coronary artery, *RA* Radial artery, *LIMA* Left internal mammary artery, *SVG* Saphenous vein graft, *RIMA* Right internal mammary artery, *DLCFA* Descending branch of the lateral circumflex femoral artery, *NA* Non-available, *p* value indicates the patency difference between the RA and non-RA grafts

Forty-five RA grafts were comprised of 43 left RAs and 2 right RAs, and these grafts included 3 Y-shaped grafts and 1 sequential graft. The distal ends of 17 RA grafts were anastomosed to the OM; 15 RAs were anastomosed to the PDA, with 10 anastomoses to the diagonal branch, and 2 and 1 anastomoses to the intermediate branch (IM) and the right coronary artery (RCA) respectively. One right RA conduit was anastomosed to the second and third OM in the sequential style; however, MDCT revealed occlusion of this vessel at the follow-up of 6 months after surgery. The short-term patency of the RA anastomosed to the left coronary artery system (LCAS) was 86.2%, which might be superior to the patency of the RA anastomosed to the right coronary artery system (RCAS) (81.3%), although the difference was not statistically significant (*p* = 0.686). (Table 4).

**Table 4:** Characteristics of 45 RA anastomoses

Category	Results	N patent	% Patent
Vessel			
Left RA	43	38	88.4
Right RA	2	0	0.0
Proximal anastomosis site			
Aorta	41	35	85.4
Left RA	2	2	100.0
LIMA	1	1	100.0
Sequential	1	0	0.0
Target vessel			
OM	17	15	88.2
D	10	9	90.0
IM	2	1	50.0
PDA	15	12	80.0
RCA	1	1	100.0

*RA* Radial artery, *LIMA* Left internal mammary artery, *OM* Obtuse marginal branch, *IM* Intermediate branch, *D* Diagonal branch, *PDA* Posterior descending artery, *RCA* Right coronary artery

## Discussion

Despite of the recommendation of current guidelines to replace the SVG with the RA in patients with high-degree coronary artery stenosis [3], few surgeons were willing to choose the RA as their preferred materials; in addition to a LIMA to the left anterior descending artery (LAD), the majority of patients received the SVG. A report from STS ACSD showed that RA grafts were used in only 6.5% of all primary isolated CABG patients in the United States between 2004 to 2015, with a gradual decrease [4]. There are several reasons for its limited utilization such as few studies reporting a mean follow-up of over 10 years and possible harm to the forearm circulation. Therefore, we describe the preliminary short-term follow-up outcomes of 41 patients who underwent CABG with the RA in order to promote the use of the RA in CABG.

Multiple studies indicated that the RA had better early-, mid- and long-term patency than the SVG, and could provide better late-term survival for CABG patients [5–8]. Recent guidelines also recommend the RA as one of the second-best arterial conduits for CABG surgery. The RA has the advantage of easy obtaining and excellent length (20-22 cm), which could reach nearly all coronary territories and result in few site-related complications [9]. In this study, no mortality events occurred without postoperative myocardial infarction or repeat revascularization events. During the follow-up, the RA had the slightly better patency rate (84.4%) than the RIMA (80.0%) and SVG (81.1%). However, due to the small sample size of this study, no statistically significant results were obtained in terms of the superiority of the RA graft; therefore, larger and longer-term clinical trials are warranted to verify whether the RA could achieve better long-term patency and patient survival compared with the SVG.

The CCB played an important role in RA patency and long-term survival. A report of Gaudino et al. showed that CABG with the RA and postoperative CCB utilization could result in better midterm results, higher RA graft patency and lower incidence of major adverse cardiac events compared with the group without CCBs [10]. Moreover, the Society of Thoracic Surgeons Clinical Practice Guidelines on Arterial Conduits recommended to use pharmacologic agents to reduce acute intraoperative and perioperative spasm for RA grafts. In this study, patients were prescribed with CCBs for 1 year after surgery. Meanwhile, surgeons should avoid touching and clamping the RA to prevent spasm during RA harvesting.

The RA anastomosed to the LCAS might provide better patency than the RA anastomosed to the RCAS. And the 2018 ESC/EACTS Guidelines on myocardial revascularization also indicated that the RA could provide better patency than the SVG, particularly for the LCAS [3]. In this study, the patency rate of RA-LCAS was 86.2%, while the patency of RA-RCAS was only 81.3%; and this characteristic of arterial grafts has been confirmed by previous literature. One explanation might be the competitive flow and different diameters of coronary arteries. Arterial grafts could autoregulate blood flow according to different metabolic needs [11–14], and the main RCA has relatively large diameter; therefore, the same stenosis degree could result in a larger residual lumen in the RCAS than the LCAS, which could provide a larger blood supply and decrease the blood flow demand from the RA. These events could cause RA graft constriction and, over time, increase the risk of RA graft atrophy and occlusion. The 2011 ACCF/AHA CABG Guideline also indicated different recommendation criteria for the application of the RA in the LCAS and RCAS; it might be reasonable to use RA grafts when grafting arteries of the LCAS with > 70% stenosis and arteries of the RCAS with > 90% stenosis [15]. Therefore, we think that the application indication of arterial conduits in the RCAS should be stricter compared with the LCAS; stenosis more than 90% of the RCAS may lead to better graft patency [7].

The forearm brachial artery and ulnar artery have good blood flow reserve capacity; after RA removal, the total blood flow of the forearm has no significant difference compared with the non-RA removal group, and RA harvesting has no significant effect on the patient’s forearm mobility and survival [7, 16–18]. In a 22-year follow-up report of Royse et al. [16], there was no difference in the total blood flow of the forearm between the RA-harvested side and the control side, and the increase of dynamic blood flow during exercise had no significant difference between the two groups. Furthermore, a 20-year follow-up study of Gaudino et al. reported the increase of the ulnar artery diameter in the operated arm without hand or forearm symptoms [7]. In this study, 2 patients developed hand paresthesia after operation, which might result from surgical trauma or ischemia neuropathy, and the hand paresthesia symptom of 1 case disappeared during follow-up.

Although ICA still represents the gold standard in the assessment of CABG graft patency, it is associated with potential complications ranging 1 to 5% [19, 20], such as bleeding, dissection, pseudoaneurysm, cardiac arrhythmia and stoke, and it is technically more difficult than MDCT in the depiction of correct anatomy of grafts, particularly in complex revascularization patients, which would undoubtedly limit its application in the routine follow-up of graft patency. MDCT could be a suitable candidate due to its noninvasive and less expensive characteristics, and it requires lower technical difficulty and shorter operating time than ICA. Moreover, MDCT could provide more additional information such as the diagnosis of early and late complications including sternal infection and pericardial or pleural effusion, the evaluation of aortic and valvular lesions, delineation of the anatomical course of bypass conduits and their topographic relationship to vital mediastinal structures; these findings could assist in the preoperative planning of redo cardiac surgery and thus reduce the incidence of graft damage.

Only a few studies evaluate CABG graft patency with MDCT, although it can bring excellent diagnostic accuracy. A systemic review of Chan and colleagues involving 1975 patients and 5364 grafts demonstrated that MDCT had the aggregated sensitivity and specificity of 98% (95% CI: 97–99%) and 98% (95% CI: 96–98%) in the assessment of bypass graft occlusion and stenosis [21]. Similarly, a study of Barbero and colleagues concluded that the diagnostic sensitivity and specificity for combined assessment of occlusion and stenosis were both over 98% [22]. Although MDCT could not replace ICA in conduit patency assessment after CABG at present, MDCT can obtain clear graft images and plays a crucial role in the evaluation of grafts, especially for asymptomatic patients. However, with future improvement of CT technologies, MDCT can provide better cardiac visualization in shorter time and might supersede ICA for graft assessment in the near future.

A limitation of this study is the relatively limited sample size and short follow-up time, compared with other larger studies. Another limitation is the lack of the assessment of graft patency with the standard reference of ICA, and there is no comparison between MDCT and ICA. However, considering the excellent accuracy of MDCT in diagnosing the patency of CABG grafts, we consider it unnecessary to correlate with ICA; and it should be stressed that the main goal of this study is to evaluate the quality of the RA, not to determine the diagnostic accuracy of MDCT.

## Conclusions

The RA has a good short-term patency rate and could lead to excellent survival outcomes. And the location of the target vessel may affect the patency rate of the RA graft; RA-LCAS might confer a better patency rate than RA-RCAS. The 128-slice MDCT scanner, providing the excellent visualization of grafts, represents a useful diagnostic tool in evaluating the quality of grafts during follow-up of CABG patients.

## Data Availability

All data generated or analyzed during this study are included in this published article.

## Abbreviations

CABG: Coronary artery bypass grafting
CCB: Calcium-channel blocker
D: Diagonal branch
DLCFA: Descending branch of the lateral circumflex femoral artery
ICA: Invasive coronary angiography
IM: Intermediate branch
LAD: Left anterior descending artery
LCAS: Left coronary artery system
LIMA: Left internal mammary artery
LVEDD: Left ventricular end diastolic dimension
LVEF: Left ventricular ejection fraction
MDCT: Multidetector computed tomography
OM: Obtuse marginal branch
PDA: Posterior descending artery
RA: Radial artery
RCA: Right coronary artery
RCAS: Right coronary artery system
RIMA: Right internal mammary artery
SVG: Saphenous vein graft
TTE: Transthoracic echocardiography
